# Target Identification for Stereotactic Thalamotomy Using Diffusion Tractography

**DOI:** 10.1371/journal.pone.0029969

**Published:** 2012-01-04

**Authors:** Zsigmond Tamás Kincses, Nikoletta Szabó, István Valálik, Zsolt Kopniczky, Lívia Dézsi, Péter Klivényi, Mark Jenkinson, András Király, Magor Babos, Erika Vörös, Pál Barzó, László Vécsei

**Affiliations:** 1 Department of Neurology, Albert Szent-György Clinical Center, University of Szeged, Szeged, Hungary; 2 International Clinical Research Center, St. Anne's University Hospital Brno, Brno, Czech Republic; 3 Department of Neurosurgery, St. John's Hospital, Budapest, Hungary; 4 Department of Neurosurgery, Albert Szent-György Clinical Center, University of Szeged, Szeged, Hungary; 5 FMRIB Centre, Department of Clinical Neurology, John Radcliffe Hospital, University of Oxford, Oxford, United Kingdom; 6 Euromedic Diagnostic Hungary Ltd, Szeged, Hungary; 7 Department of Radiology, Albert Szent-György Clinical Center, University of Szeged, Szeged, Hungary; Bellvitge Biomedical Research Institute-IDIBELL, Spain

## Abstract

**Background:**

Stereotactic targets for thalamotomy are usually derived from population-based coordinates. Individual anatomy is used only to scale the coordinates based on the location of some internal guide points. While on conventional MR imaging the thalamic nuclei are indistinguishable, recently it has become possible to identify individual thalamic nuclei using different connectivity profiles, as defined by MR diffusion tractography.

**Methodology and Principal Findings:**

Here we investigated the inter-individual variation of the location of target nuclei for thalamotomy: the putative ventralis oralis posterior (Vop) and the ventral intermedius (Vim) nucleus as defined by probabilistic tractography. We showed that the mean inter-individual distance of the peak Vop location is 7.33 mm and 7.42 mm for Vim. The mean overlap between individual Vop nuclei was 40.2% and it was 31.8% for Vim nuclei. As a proof of concept, we also present a patient who underwent Vop thalamotomy for untreatable tremor caused by traumatic brain injury and another patient who underwent Vim thalamotomy for essential tremor. The probabilistic tractography indicated that the successful tremor control was achieved with lesions in the Vop and Vim respectively.

**Conclusions:**

Our data call attention to the need for a better appreciation of the individual anatomy when planning stereotactic functional neurosurgery.

## Introduction

Thalamotomy was introduced in the treatment of tremor by Hassler in 1954 [1], the selective stereotactic lesioning of the ventralis intermedius nucleus of the thalamus (Vim) was described by Narabayashi [2] and later the electrical stimulation of Vim in 1987 by Benabid [3]. The precise targeting within the brain is of crucial importance for successful surgical intervention. Targeting the desired thalamic nucleus is usually carried out by using stereotactic coordinates specified in relation to a point on the anterior commissure – posterior commissure (AC-PC) line [4], [5], [6]. Other methods try to establish population-based stereotactic coordinates of the target nuclei based on postmortem histological data or intraoperative stimulation techniques [7], [8], [9]. While achieving reasonable results, these methods neglect individual anatomical variations.

Since the identification of thalamic nuclei on conventional imaging modalities is difficult, several novel approaches were proposed to aid the visualisation of the functionally important thalamic nuclei. It is now possible to segregate the major thalamic structures using MR relaxometry, even in a clinically acceptable time [10], [11]. Recently, probabilistic tractography was successfully used to investigate the connectivity profile of two major thalamic target nuclei for functional neurosurgery: ventralis intermedius (Vim) and ventralis oralis posterior (Vop) [12]. In addition, it is possible to segment the thalamic nuclei based on connectivity patterns defined by MR diffusion tractography [13], [14]. This approach has been suggested by others [12], [15], [16], but the benefit of such a method has not been evaluated systematically so far.

In the current investigation we calculated the interindividual variability of the target thalamic nuclei, (Vop and Vim) location in healthy controls. We also present the retrospective DTI identification of the target thalamic nuclei in two patients that underwent stereotactic Vim and Vop thalamotomy.

## Methods

### Participants

Nine healthy individuals, with no history of neurological or psychiatric diseases were included in the study (mean age: 28.3±7.09, male: 3). Furthermore, two patients were also included, who underwent stereotactic thalamotomy.

### Ethics

The study was approved by the Ethics Committee of University of Szeged (authority number: 87/2009), and all subjects provided written consent.

### Image acquisition

Imaging was carried out using a 1.5 T GE Signa Excite scanner. High resolution T1 weighted images (3D IR-FSPGR: TR/TE/TI: 10.3/4.2/450ms, flip angle: 15°, ASSET: 2, FOV: 25*25 cm, matrix: 256*256, slice thickness: 1mm) and diffusion-weighted images (DTI: TR/TE: 16000/93.8ms, flip angle: 90°, FOV: 23*23cm, matrix: 96*96, slice thickness: 2.4mm, ASSET: 2, NEX: 2) were acquired. Diffusion weighting was performed along 60 independent directions, with a *b*-value of 1000 s/mm^2^. Six reference images, with no diffusion weighting were also obtained. The preoperative scans of the patients were obtained using identical acquisition sequences to those used for control subjects. Postoperative scans were carried out three months after the surgery. High-resolution-T1 weighted images (with parameters identical to the preoperative ones) and sagittal 3D FLAIR images (3D FLAIR: TR/TE/TI: 6000/134.6/1839ms, flip angle: 90°, FOV: 23*23cm, matrix: 256*256, slice thickness: 2mm) were acquired to localise the thalamotomic lesion.

### Image processing

Data were processed using the tools from the FMRIB Software Library (FSL, version 5.0; Oxford Centre for Functional MRI of the Brain (FMRIB), UK; www.fmrib.ox.ac.uk/fsl [17]) according to the method reported by Behrens and colleagues [14]. Initially the raw diffusion data was corrected for eddy-currents and motion artefacts with FMRIB's Diffusion Toolbox (FDT [18]). The images were then skull stripped using the Brain Extraction Tool (BET [19]) and the diffusion-weighted images were registered to the high-resolution T1-weighted image with a 6 degree-of-freedom linear registration using the FMRIB Linear Image Registration Tool (FLIRT [20]). Probability distributions of fiber orientation were estimated for each brain voxel in the acquired diffusion space using a multi-fiber extension [21] of the probabilistic tractography available in FDT [18].

Probabilistic diffusion tractography can estimate the pathways that originate at any given seed voxel, as well as the probability that such a pathway will pass through any other voxel in the brain [18]. Binary masks of the thalamus and the cortical targets were drawn manually for each subject. Probabilistic multi-fiber diffusion tractography was initiated from every voxel inside the thalamic mask: 5000 times for each voxel. Counters were increased every time an individual streamline reached the cortical target region. Hence, the values stored in thalamic voxels in one of the resulting images represent the probability of those voxels being connected to the particular target cortical mask assigned to that image [14].

To investigate the inter-subject variability of the target nuclei, a specific distance reserving registration method was used: the high-resolution T1-weighted images were registered to the standard MNI brain with a 6 degree-of-freedom transformation (rotations and translations only) that was based on landmark points in a way that kept the position of the anterior commissure fixed, while aligning the ACPC line and the mid-sagittal plane between images.

The Euclidean distance, given by the equation:
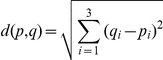



was calculated for each subject between points representing the thalamic voxel with peak connection probability to the premotor cortex (for Vop) and to the motor cortex (for Vim) and the standard space voxel with peak connectivity as indicated by the Oxford Thalamic Connectivity Map [13]. The mean pair-wise distance was also calculated: the distance between the voxel with peak connection probability to the premotor (for Vop) and to the motor (for Vim) cortex was calculated between all possible pair of subjects and this inter-subject distance was averaged for Vop and Vim separately.

The connectivity images for the thalamus were thresholded at 10% of individual connectivity maximum and binarised in order to create masks of the thalamic regions connected to the premotor or motor cortex. To assess the similarity of positions the overlap between each pair of masks (between subjects) were calculated according to the method proposed by Crum and colleagues [22]. Overlap was measured by the Tanimoto Coefficient (TC), which is defined as the ratio of the number of voxels in the intersection of the two regions to the number of voxels in the union:



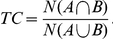



We also took further steps to exclude possible registration bias between the DTI and T1-weighted images that might originate from the EPI distortions. We manually compared the high-resolution T1-weighted image and the first volume of the diffusion data (no diffusion gradient applied) registered to the T1-weighted image for each subjects. The positions of the following landmarks were compared: AC, PC, the maximal width of the third ventricle at the level AC and the highest point of the corpus callosum in the midsagittal line.

## Results

### Inter-subject variability of the position of thalamic nuclei

As found with the manual comparison the shape and the position of the thalamus was not affected by EPI distortions. The largest misregistrations were found along the anterior-posterior axis, but even that was minimal (for AC and for PC 0.6±0.5mm). The width of the third ventricle was not different, and the position of the highest point of the corpus callosum differed only in case of a single subject with 1 mm.

**Figure 1:**
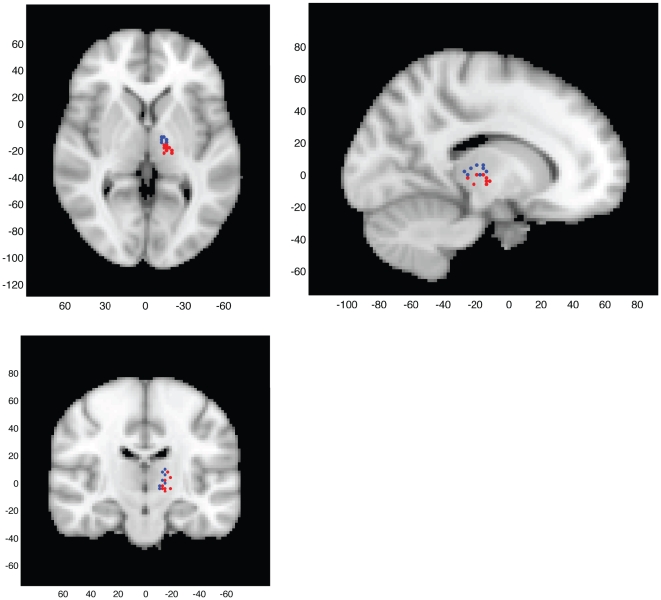
Spatial localisation of peak Vop and Vim voxels as defined by probabilistic tractography. Individual images were realigned to standard space with a 6 DOF transformation in a way to match the location of the AC and align the AC-PC line and the midsagittal plane. The registered peak connectivity voxels for the primary motor (Vim - in red) and for the premotor cortex (Vop – in blue) are shown on three orthogonal slices.

The premotor thalamus was consistently localised in all subjects, however, since the individual brain shapes differed, the exact location of the Vop and Vim nucleus varied substantially across subjects (Fig 1). The mean distance of the peak connection probability of Vop from the peak probability indicated by the Oxford Thalamic Connectivity map was 5.08mm. The equivalent distance was 6.26mm for the Vim. The mean pair-wise inter-subject distance of the peak connectivity voxel of Vop was 7.33±3.37mm (range: 0–14.56mm) and 7.42±3.35 mm (range: 2–14.28mm) for Vim. The mean distance of the tractography defined Vop and Vim coordinates from the Stereotactic target point defined by Hyam's method [12] was 7.19±4.36mm (range: 2.45–14.89mm) for Vim and 9.58±4.82mm (range: 3.0–17.12mm) for Vop.

The mean pair-wise overlap for Vop as calculated by the Tannimoto Coefficient was 40.2% (range: 15.5%–66.2%) and 31.8% (range: 3.2%–66.2%) for Vim.

### Case #1: Vop thalamotomy for posttraumatic tremor

**Figure 2:**
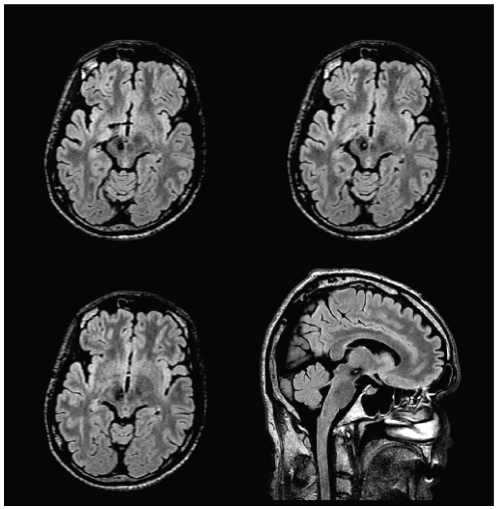
Preoperative FLAIR images show the right perirubral mesencephalic traumatic haemorrhage. Images are presented in radiological convention.

A left-handed, 32 year-old male, after having a motorbike accident that caused brainstem bleeding (at the level of the red nucleus; Fig 2), developed a tremor having resting, postural, as well as intention components, bradykinesia, rigidity and a slight paresis of the left arm. Seven years after the accident, because of ineffective tremor control by various medication regimes, functional neurosurgery was offered. Because the patient has not accepted deep-brain-stimulation implantation stereotactic thalamotomy was carried out on the right side in the region of Vop/Vim border with target coordinates according to Guiot's method [5], [23] that is, given the AC-PC length of 24.9mm, was 8mm anterior of PC and 12.7mm lateral from the mid-sagittal line on the AC-PC plane. During the standard surgical procedure the final position of the lesion along the selected trajectory was adjusted according to the tremor control of the intraoperative stimulation. After surgery the patient had an immediate relief from his left upper extremity tremor (his score on the part A of the Fahn-Tolosa-Marin tremor rating scale (FMT) reduced form 16 to 4 points). Neither the muscle strength, nor the bradykinesia improved. At the three-month checkup these findings had not changed for the left upper extremity, however a small amplitude flexion-extension tremor appeared in the left toes.

**Figure 3:**
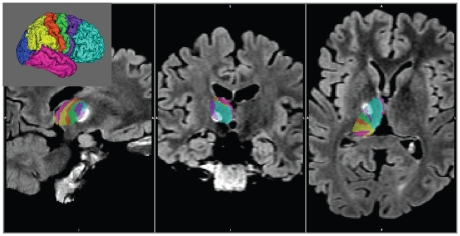
Connectivity-based segmentation of the right thalamus of the patient operated on for tremor caused by traumatic mesencephalic bleeding. Results are overlaid on the postoperative FLAIR images. The bright area represents the Stereotactic thalamotomy. The key to the colour coding of the thalamic segmentation is shown in the inset. The colour in each thalamic voxel represents the colour of the cortical area that has the highest connection probability to that voxel.

As shown by the connectivity-based segmentation of the thalamus, regions connected with highest probability to the prefrontal, premotor, motor and sensory, parietal, occipital and temporal cortices were clearly delineated (Fig 3). The operative lesion of the thalamus, identified on the postoperative FLAIR images (registered to the preoperative T1-weighted structural image with 6 DOF linear registration) was situated in the region of the thalamus connected with the highest probability to the premotor cortex (Fig 3).

**Figure 4:**
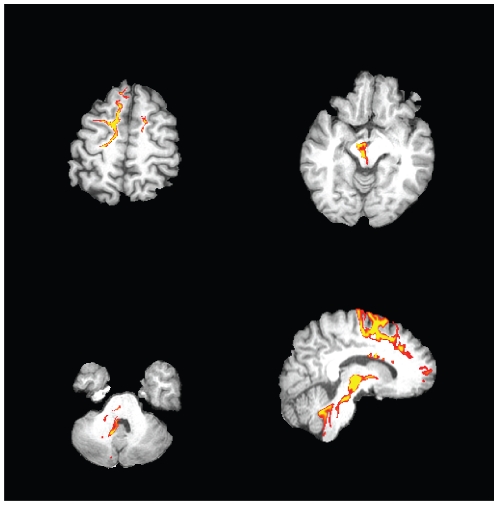
Connectivity of the thalamic lesion, evaluated using multi-fiber probabilistic tractography. The tracts reach the premotor cortex, on one hand, and the nucleus ruber and the perirubral mesencephalon, on the other hand, with some travelling further to the cerebellum. The image is thresholded at 500 particles (10%).

Probabilistic multi-fiber tractography initiated from the thalamic lesion showed, on one hand, connection to the medial premotor - prefrontal cortex and, on the other hand, to the brain stem particularly to the rubral/perirubral mesencephalic posthaemorrhagic lesion and some fibres travelling further on to the cerebellum (Fig 4).

### Case #2: Vim thalamotomy for essential tremor

A right-handed, 50-year-old women had developed tremor ten years earlier. The tremor was most prominent in the hands but also appeared in the head, having postural and action components, and was also present when walking. Her family history was unremarkable. Medication regimes that had been tried over the years were ineffective, and the tremor severely affected the quality of life of the patient. Stereotactic Vim thalamotomy was carried out with target coordinates according to Guiot [5], [23] that correspond well with the coordinates defined by Hyam's method [12]. After surgery the tremor of the right hand improved significantly and on the three-month control examination this effect was found to be enduring (FMT score reduced from 17 to 11 points). No significant change was detected in the head-tremor or in the tremor of the left hand.

**Figure 5:**
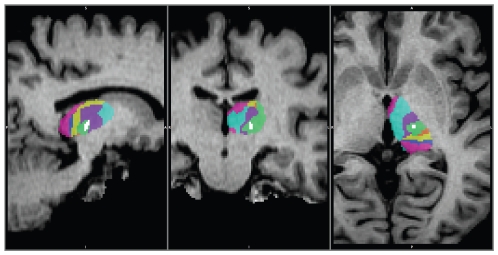
Connectivity-based segmentation of the left thalamus of the patient having essential tremor. Results are overlaid on the postoperative T1-weighted image. White voxels represent the location of the Stereotactic lesion. The colour coding of the thalamic segmentation is the same as that using in Figure 3. The lesion is in the area of the thalamus connected most strongly to the primary motor cortex: the putative Vim.

**Figure 6:**
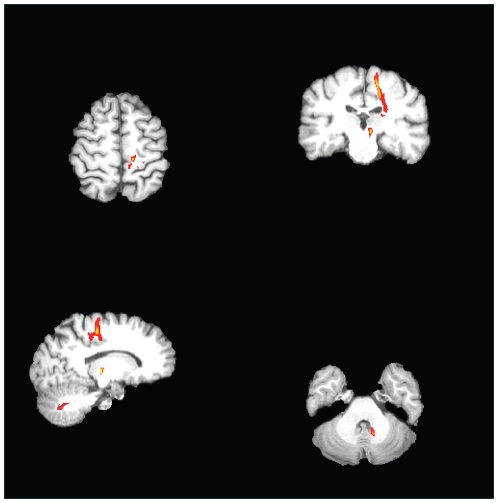
Connectivity of the thalamic lesion of the essential tremor patient, evaluated using multi-fiber probabilistic tractography. The tracts reach the primary motor cortex, on one hand, and travelling to the cerebellum on the other hand. The image is thresholded at 250 particles (5%).

The postoperative T1-weighted image was registered to the preoperative T1-weighted image in order to identify the position of the lesion in relation to the diffusion–based connectivity results. The connectivity-based segmentation of the thalamus indicated that the surgical lesion was in the putative Vim nucleus, the region that showed the highest probability of connection to the primary motor cortex (Fig 5). Postoperative probabilistic tractography from the lesion showed that the lesioned thalamus was connected to the primary motor cortex, mainly to the medial structures, and downstream to the ipsilateral cerebellar hemisphere (Fig 6).

## Discussion

In the current study we investigated the inter-individual variability of the position of the thalamotomic target nuclei Vim and Vop as defined by probabilistic diffusion tractography. We found that, compared to the size of the nucleus, the variability of its position is substantial. Our results call attention to the importance of defining the thalamotomic lesion target individually. We also showed that the lesion in two successful thalamotomic cases was in fact in the target nuclei as defined by tractography based segmentation.

Despite the accurate stereotactic atlases [7], [24] that guide the standard methods for targeting, significant intersubject variability still exists [7]. The differences in gross thalamic morophology is already conspicuous: the medio-lateral aspect as defined by the position of the internal capsule, as well as the height of the thalamus vary between subjects [7]. The position of the pulvinar system varies even between subjects with the same AC-PC distance [7]. Moreover, as emphasized by Morel, these variations are not even homogenous within the thalamus, particularly along the mediolateral axis [7]. Our results, using a different modality, also indicate significant variability of the position of the Vop and Vim nuclei, especially when the size of the nucleus and the surgical lesion is considered. The distance between peak-probability Vop voxels was 7 mm on average and is similar for Vim, but in certain cases the distance was as much as one and a half centimetres. The overlap between Vops on average was around only 40% and varies from 15% to 60%. Similar overlap values were found for Vim. It is also important to emphasize that in the current variability estimation only young, healthy individuals were included. Brain pathology may further increase the variability.

One possible caveat of our analysis that cannot be neglected is the misregistration when aligning the high resolution T1-weighted and diffusion weighted images. The diffusion-weighted images suffer significant distortions because of susceptibility artefacts, however our analysis indicated that this distortions was minimal in the region of thalamus.

The high inter-subject variability should be accounted for during surgery. The first approach is to standardise the position of the lesion according to internal reference distances such as the length of the AC-PC line, the height of the thalamus and the width of the third ventricle [5], [24]. Instead of using the histology-based stereotactic atlas derived from a limited number of brains, recently a population based-probabilistic functional atlas was developed from data gathered from pre-, intra- and postoperative neuroimaging and electrophysiological investigations [8], [9]. The extension of this approach is the physiological control of lesion depth during awaken surgery by monitoring patients complains while trying different stimulation positions.

It follows from the discussion above that, ideally, individual anatomy should be respected when performing stereotactic thalamotomy. Recently, Yamada and colleagues suggested a diffusion-based method for identification of the ventral thalamic nucleus [15]. Their approach incorporates tracking of the cerebello-thalamo-cortical and the spino-thalamic tracts that cross the thalamus, and the identification of anatomical landmarks on short tau inversion recovery (STIR) and fractional anisotropy images [15]. Sedrak and co-workers used the fractional anisotropy weighted color-coded diffusion direction maps to manually segment the thalamus from the surrounding white matter and identify the ventral intermedius nucleus [16] to define target for deep-brain-stimulation (DBS). The connectivity of effective electrode location investigated by tractography showed connections to the sensory-motor cortex and to the cerebellum. Compared to these deterministic approaches our method offers further advantages by utilising probabilistic tractography [14], [18]. This method is able to give statistical evaluation of connection probabilities to cortical targets for every thalamic voxel that - by appropriate selection of the cortical targets – are then able to delineate thalamic regions that correspond well to thalamic nuclei as defined by histology [14], [18], [25]. This approach shows a high intra-subject reproducibility in terms of repeated analysis as well as repeated data acquisitions [25].

Within the ventral thalamic nuclear complex the ventralis oralis anterior/posterior (Voa/Vop) and the ventralis intermedius nucleus (Vim) can be delineated. The true connectivity of these nuclei is debated, and it is hard to mach different nomenclatures [26], but as a rule of thumb it can be said that Vim is the cerebellar receiving area while Vop is the pallidal receiving area of the thalamus [27], [28]. More importantly, from our point of view, cortical connectivity of the putative Vim and Vop was recently investigated using diffusion imaging [12]. It was found that Vop seeded tractograms connected to the supplementary motor area (SMA) and to the dorsolateral prefrontal cortex (DLPFC) with higher probability than Vim-seeded tractograms; while the Vim was more likely to connect to the primary motor cortex (M1) and contralateral cerebellum, although significant cerebellar connections were found for Vim as well as for Vop [12]. Here we provide a confirmation of Hyam's pioneering analysis by showing that the Vop and Vim coordinates selected for thalamotomy were indeed effective. The connectivity profile of our patients' thalamotomic lesions were strikingly similar to those described as Vop and Vim connectivity profiles by Hyam [12].

Lesioning or stimulation of the ventral thalamic nuclei is a frequently considered option for the treatment of tremor of various causes including post-traumatic Holmes tremor [29], [30], [31], [32], [33], [34]. Within the ventral nuclei, Vim is the optimal target in essential tremor [35], [36]. It is less and less frequently used in the treatment of Parkinsonian tremor because of the poor benefits for bradykinesia and rigidity [37]. In multiple sclerosis, beside targeting Vim some authors prefer to target Vop suggesting better control of the ataxic component of the tremor [4], [38]. In tremor cases developed after injury of the superior cerebellar peduncules or the rubrocerebellar connections (e.g. traumatic brain injury, tumour) tremor control usually is difficult. Reports about the stimulation/lesion of the Vim [32], [33], [39] and Voa/Vop [40] indicated positive outcome. Foote and colleagues also reported three posttraumatic tremor cases and one MS tremor case that responded to dual Vim-Voa/Vop stimulation [41]. Based on the connectivity profile, the thalamic lesion in our patient was in the Vop. This lesion resulted in excellent tremor control, however the rigidity and the bradykinesia of the affected extremity did not improve significantly. It also has to be mentioned that probabilistic tractography was recently also successfully used in defining connectivity of the pedunculo-pontine nucleus, a novel target for neurosurgical treatment of Parkinson's disease [42].

### Conclusions and limitations

Atlas-based targeting for stereotactic functional neurosurgery is a frequently considered option for the treatment of movement disorders. While the overall outcome is reported to be positive, the failures and side effects might be related to the unaccounted inter-individual variability of the target localisation. To overcome such limitations we suggest incorporating information about individual anatomy from diffusion imaging, using connectivity-based segmentations of the thalamic nuclei. However, it has to be pointed out that in the two cases we reported in current analysis we did not plan the targeting with tractography based segmentation a priori of the surgery. Extension of this study to compare the result of the stereotaxic neurosurgery with and without tragtography based targeting is crucial. Further improvements might be expected if additional imaging modalities (e.g. relaxometry, fMRI) could also be utilised.
